# A Systematic Review of Salt Taste Function and Perception Impairments in Adults with Chronic Kidney Disease

**DOI:** 10.3390/ijerph191912632

**Published:** 2022-10-03

**Authors:** Sze-Yen Tan, Paridhi Tuli, Giecella Thio, Breannah Noel, Bailey Marshall, Zhen Yu, Rachael Torelli, Sarah Fitzgerald, Maria Chan, Robin M. Tucker

**Affiliations:** 1 School of Exercise and Nutrition Sciences, Deakin University, 221 Burwood Highway, Burwood, VIC 3125, Australia; 2 Institute for Physical Activity and Nutrition (IPAN), Deakin University, 221 Burwood Highway, Burwood, VIC 3125, Australia; 3 Department of Nutrition and Dietetics, The St. George Hospital, Kogarah, NSW 2217, Australia; 4 Faculty of Science, Medicine and Health, University of Wollongong, Wollongong, NSW 2522, Australia; 5 Department of Food Science and Human Nutrition, Michigan State University, 2110 Anthony Hall, 474 S Shaw Lane, East Lansing, MI 48824, USA

**Keywords:** chronic kidney disease, salt taste, thresholds, intensity, liking, preference

## Abstract

Individuals with chronic kidney disease (CKD) experience physiological changes that likely impair salt taste function and perception. Sodium restriction is a cornerstone of CKD management but dietary sodium plays an important role in food enjoyment and may interfere with compliance to this intervention. Therefore, confirming that taste deficits are present in CKD will improve our understanding of how taste deficits can affect intake, and inform dietary counselling in the future. A systematic review was conducted. Studies that included adults with CKD and healthy controls, and assessed salt taste sensitivity, perceived intensity, and/or hedonic ratings were included. Study quality was assessed using the Academy of Nutrition and Dietetics Evidence Analysis Library Quality Criteria Checklist: Primary Research. Of the 16 studies, the majority reported decreased salt taste sensitivity, but no consistent differences in intensity or hedonic ratings were observed. Higher recognition thresholds in CKD patients were associated with higher sodium intake, but results should be interpreted with caution as the measures used were subject to error in this population. In conclusion, salt taste sensitivity is decreased in CKD, but intensity and hedonic evaluations appear to be more robust. Given that hedonic assessments are better predictors of intake, and that salt taste preferences can be changed over time, dietary counselling for low-sodium intake is likely to be effective for this population.

## 1. Introduction

In 2017, chronic kidney disease (CKD) was estimated to affect 9.1% of the global population [1]. CKD is a chronic condition characterised by damage to the structure and function of the kidneys, and the build-up of waste products in the body, which cause other health problems [2]. CKD progresses in five stages, ranging from a normal/high glomerular filtration rate (GFR) of >90 mL/min to end-stage kidney disease (GFR < 15 mL/min) [3]. In the advanced stages of CKD, individuals will require dialysis or kidney transplant to remove waste products and maintain electrolytes and fluid balance.

Hypertension is a common etiology of CKD, and renal insufficiency, in turn, results in water retention in the body and subsequently increases blood pressure [4]. For this reason, one of the primary goals of CKD management is to manage blood pressure in order to prevent the further progression of this disease and co-morbidities, such as cardiovascular disease, and to improve prognosis [4,5,6,7]. Several strategies are used in the management of the blood pressure of individuals with CKD, one of which is the control of dietary sodium intake [8]. Restricting sodium intake is necessary as consumption stimulates thirst, promotes water intake, and increases water retention and blood pressure; hence, high sodium intake can further exacerbate the CKD condition [9], including proteinuria and albuminuria [7].

While reducing sodium intake in individuals with CKD is essential, adherence to this dietary recommendation is often poor [10]. Sodium provides a pleasant salty taste to foods and enhances food flavour and palatability, and sodium restriction affects eating enjoyment [11]. Furthermore, there is evidence that individuals with CKD may experience taste changes, including impaired salt taste sensitivity, which was self-reported in as many as 58% of individuals with CKD [12].

Based on the available evidence, a number of CKD-related histological and physiological changes offer insights on potential mechanisms of taste impairments in this population. First, fewer taste buds (where taste receptors are found) have been observed in individuals with CKD [13]. Second, the production of saliva, which dissolves and delivers tastants to taste buds [14], is also reduced in CKD [15,16,17]. Third, studies have reported increased salivary sodium and potassium concentrations, which may desensitise individuals with CKD to the salt taste and amplify the metallic taste [16,18,19]. A potential implication of higher salivary mineral concentrations is that higher concentrations of sodium from the diet are needed to be detected and recognised, as well as to be perceived as pleasant. Fourth, high blood urea levels in CKD increase salivary urea levels; the breakdown of salivary urea increases salivary pH, which can interfere with sour taste perception [18,20]. As urea is a bitter tastant, a heightened salt taste preference or sodium intake may occur in order to counter this bitter taste [21]. Fifth, zinc deficiency is prevalent in individuals with CKD, which can lead to low gustin (a growth factor of taste buds) and, subsequently, taste impairment [22]. Zinc plays an important role in taste function as high zinc levels or zinc supplementation were demonstrated to reverse impaired taste sensitivity [23,24].

Although the histological and physiological changes [25] associated with CKD appear to support the high likelihood of taste changes in individuals with CKD, it is still unclear if these abnormalities, in fact, translate into objectively measurable taste impairments given the importance of sodium restriction in CKD management. This question forms the basis of this systematic review: to test the hypothesis that individuals with CKD have impaired salt taste function and perception compared with healthy adults without this condition.

## 2. Materials and Methods

### 2.1. Search Strategy

A systematic literature search was conducted using four online databases: PsycINFO, Embase, PubMed and CINAHL. This search included all publications that were available until 25 February 2022. Three main search themes were used. The first theme was related to kidney disease, where we included the keywords: kidney disease OR CKD OR chronic kidney disease OR renal failure OR renal insufficiency OR end-stage renal failure OR ESRF OR end stage renal failure OR end-stage renal disease OR end stage renal disease OR ESRD or nephron*. The second theme was related to taste function and perception, where keywords included: taste sensitivity OR taste preference OR taste function OR threshold OR sensitivity OR intensity OR liking OR prefer*. The third theme was related to specific salt taste quality and keywords included: salt OR salty. Filters were applied so that publications were studies that recruited human participants and published in English only. A snowball search was conducted based on the reference list of relevant publications identified during the systematic search. The protocol of this systematic literature review was registered with PROSPERO, reference No. CRD42022295333.

### 2.2. Study Screening and Selection

The search results from all four databases were exported into Covidence, a platform where all duplicated publications were removed. Using the inclusion and exclusion criteria, two researchers independently screened the titles and abstracts of the publications to remove those that were not relevant to this review. Both researchers were required to come to the same decision. Any conflicts were discussed between the two researchers and, if they remained unresolved, a third researcher intervened, and a decision was made. In the next step of the study selection, a full-text screening by two researchers was again independently conducted, and the same conflict resolution approach was utilised. The reasons for study exclusion at the full-text screening stage were documented and categorised into four exclusion groups, namely incorrect study population, incorrect study design, incorrect outcome studied or incorrect intervention.

### 2.3. Eligibility Criteria

Studies were deemed eligible if they included adults (18 years and over) with CKD and assessed salt taste objectively as salt taste sensitivity (detection and recognition thresholds), perceived salt taste intensity, or liking of salt taste, or preferred salt taste concentrations. In this review, the salt taste detection threshold is defined as the lowest concentration of a salty tastant to be reliably detected and differentiated from a control (e.g., water), while the recognition threshold refers to a concentration where an individual is able to not only detect the stimulus but also to recognise it as salt taste. Significantly higher detection and/or recognition thresholds would indicate lower salt taste sensitivity. All articles were scholarly peer-reviewed articles, with a full-text version available for review. For the purpose of this review, we included studies that recruited adults with all stages of CKD, who were or were not undergoing treatments such as dialysis (with the exception of renal transplantation), and included a comparator group who were healthy adults (between-subject study design) or the same participants before and after CKD diagnosis (within-subject study design). A meta-analysis was not possible due to the heterogeneity in CKD stages/treatment and testing procedures.

### 2.4. Data Extraction

Relevant data from all articles were extracted by two researchers. The information extracted included: author, year of publications, stages of CKD, form and duration of treatment if any, e.g., haemodialysis and peritoneal dialysis such as continuous ambulatory peritoneal dialysis (CAPD) or automated peritoneal dialysis (APD), age/sex of participants, methods used to assess salt taste function and perception, key findings, and any other outcomes relevant to salt taste function and perception, such as other taste qualities (sweet, bitter, sour, or umami), as well as dietary intake since taste is often cited as a primary driver of intake.

### 2.5. Assessment of Study Quality

Using the Academy of Nutrition and Dietetics Evidence Analysis Library Quality Criteria Checklist: Primary Research, all articles underwent a critical appraisal [26]. The checklist consists of four “relevance” questions and ten “validity” questions, which assess the quality of the publications. The “relevance” questions consider if the intervention would result in improved outcomes for the target population and if the outcome is important to the targeted population. The “validity” questions were answered by assessing the risk of selection and attrition bias; the implementation of blinding, if any intervening factors were described; the reliability of the measurement tools used; how appropriate the statistical analysis was; and if there were any conflicts of interest due to funding or sponsorship. The overall quality rating for the article was determined positive if “yes” was the answer for validity questions 2, 3, 6, and 7 and at least one additional question was met. A neutral rating was applied if some of the criteria for the validity questions were met, and a negative rating was given if most of the criteria for the validity questions were not met.

## 3. Results

### 3.1. Search Results and Study Quality

**Figure 1:**
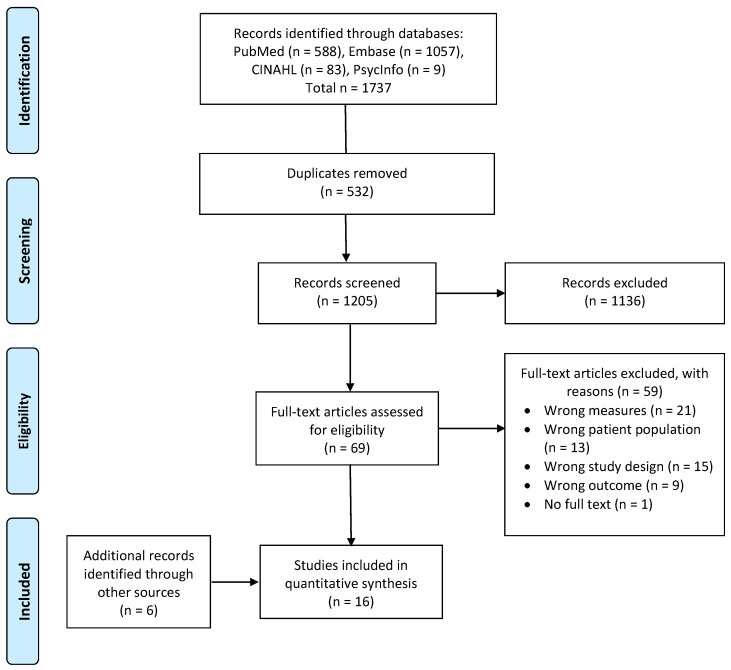
PRISMA flowchart.

Figure 1 shows the database search results and publication selection process. The initial literature search from four databases yielded 1737 publications. After removing duplicates, a total of 1205 articles remained for screening. During the title and abstract screening, 1136 publications that did not meet the inclusion criteria were excluded. The remaining 69 full-text articles were screened, of which 10 publications that met the inclusion criteria were included [27,28,29,30,31,32,33,34,35,36]. Six articles were further identified via snowball search [37,38,39,40,41,42], leading to a total of 16 publications, representing 16 unique studies. From the 16 studies, twelve were assigned a “positive” quality rating as detailed within Table 1, and the remaining four articles were assigned as “neutral”.

### 3.2. Study Characteristics

Fifteen of the sixteen studies were observational studies (14 cross-sectional [27,28,29,30,31,32,33,34,35,36,37,38,39,42] and a case–control study [41]). The remaining experiment was an interventional study [40], but only the pre-intervention baseline data were included in this review to eliminate the effects of intervention on the salt taste function of individuals with CKD. All studies included individuals with various CKD stages, and some, but not all, of the participants were undergoing haemodialysis (HD) [28,32,36,37,38,39,40,42] or continuous ambulatory peritoneal dialysis (CAPD) [32,34,37,39,42]. The studies included in this review were conducted in several countries, and they varied in terms of sample size, ranging from 28 to 510 participants in total (see Table 2). Healthy adults were included as controls/comparators in all studies, four studies made the effort to match the age, sex, or body mass index (BMI) of healthy individuals and those with CKD [27,37,40,41]. The 16 studies included in this review included at least one measure of salt taste function and perception, namely detection threshold, recognition threshold, perceived intensity, and hedonics, as shown in Table 2.

### 3.3. Detection Threshold, Recognition Threshold, and Perceived Salt Taste Intensity

Of the four studies that measured detection thresholds (DT) (Table 3), three studies [29,30,34] reported significantly higher DT in individuals with CKD compared with healthy adults. The absence of CKD-related salt taste impairment in the remaining study [36] did not appear to be attributable to sample size, CKD treatment, or taste test protocol. In terms of recognition thresholds (RT), six out of ten studies reported significantly a higher RT (hence, less sensitivity) to salt taste in the CKD group [28,29,30,33,37,41]. As with the studies of DTs, there were no obvious reasons why the remaining four studies failed to observe significant differences between individuals with CKD and healthy adults’ salt taste RT.

At concentrations above DT and RT, also known as “suprathreshold” concentrations, the perceived intensity of a taste is also an indicator of taste sensitivity. A total of six studies assessed the intensity ratings of salt taste stimuli (Table 3). Five out of six studies failed to observe significant differences between the intensity ratings of salt taste stimuli between individuals with and without CKD [20,32,33,39,40]. In the only study that did report differences, salt taste intensity ratings were significantly higher among those with CKD [38] but only after data were adjusted for how the participants had rated the intensity of “water” (as control). This statistical adjustment was not performed in the abovementioned five studies that found no difference. This difference in analysis suggests the possibility that the data analysis approach may influence outcomes. That said, this adjustment is not common practice in the field. To summarise, despite the evidence that suggests individuals with CKD were less sensitive to salt taste (higher DT and RT), this impairment did not translate into differences in perceived salt taste intensity.

### 3.4. Salt Taste Hedonics

Participants’ hedonic ratings towards saltiness were assessed in six studies: two studies assessed preferred salt concentrations in soup [29,40], two asked participants to rate their liking of salt (NaCl) solutions [31,38], and two studies assessed participants’ preference for a list of salty foods (Table 3) [39,42]. Findings were mixed, where three studies found a significant difference in salt taste hedonics between individuals with CKD and healthy individuals [29,38,39], while the remaining three did not [31,40,42]. Inconsistent findings were even found between the three studies that reported significant differences; for example, individuals with CKD were found to prefer salty foods more [39], dislike salty solutions less [38], or prefer lower salt concentrations in their soup [29]. Therefore, a conclusion regarding hedonic perception could not be drawn because of the conflicting findings.

### 3.5. Other Relevant Findings

In addition to the primary aim of this review, to investigate the relationship between CKD and salt taste function and perception, we further explored whether taste impairment occurred in other taste qualities, and whether salt taste impairment correlated with sodium intake. In terms of other taste qualities, 11 studies also performed evaluations of sweet, sour, bitter, and/or umami tastes (Table 2 and Table 3). These studies also reported impairment in other taste qualities, albeit not consistently. Overall, these studies reported decreased sensitivity to, or a reduced ability to recognise, sweet [27,28,32,41], sour [27,28,31,41], bitter [28,31,34,35,37,41], and umami [28,31,32] tastes. The only exception was one study where individuals with CKD were found to have significantly lower DTs (more sensitive) for sweet, sour, and bitter than healthy adults [36]. Some studies also documented significantly lower intensity ratings on sour [31,32,33], bitter [31], and umami [33] tastes. Therefore, an impairment in taste sensitivity was observed across multiple taste qualities rather than being limited to salt taste only.

Since taste is often cited as an important driver of dietary intake, we further extracted findings on salt taste function and preference and dietary sodium intake from these studies. Only four studies in this review examined the relationship between salt taste function and dietary sodium intake, or its proxy measurements, such as urinary sodium excretion. Salt taste DT did not correlate with urinary sodium excretion in the two studies that included these measures [29,30]. Salt taste RT was found to positively correlate with the urinary sodium excretion of individuals with CKD (r = 0.57, *p* < 0.01) [30]. A larger proportion of participants with CKD who were able to recognise salt and umami tastes correctly had a sodium intake within the recommended levels [33]. In contrast, another study did not observe a significant correlation between salt taste RT and urinary sodium excretion [29]. Finally, the preferred salt concentration in soup was positively associated with urinary sodium excretion (beta = 0.17, *p* = 0.022), although this study actually found a lower preference for salt in individuals with CKD than healthy adults [29]. In other words, there is a possibility that altered salt taste function (mainly higher RT) in individuals with CKD may translate into actual sodium intake. However, several limitations related to dietary assessment and potential confounding factors were noted (discussed in the next section); hence, the findings should be interpreted with care.

## 4. Discussion

The aim of this review was to explore whether taste function and perception in adults diagnosed with CKD differs from those without, and how these measures are associated with dietary intake. Based on the 16 studies included in this systematic review, the majority reported an impairment in salt taste sensitivity, i.e., the ability to detect and recognise salt taste. However, there was no evidence that individuals with CKD perceived salt taste to be less intense, and findings were mixed in terms of their hedonic ratings of saltiness.

Purely from a sensory science perspective, increased detection and recognition thresholds in individuals with CKD imply that higher concentrations of salt in foods are needed to achieve the same level of stimulation in healthy adults. Therefore, a hypothesised implication of impaired salt taste sensitivity in individuals with CKD is that they may end up consuming more sodium, the most common salt tastant in our diet, which can result in a range of consequences that interfere with CKD management, such as thirst and excessive water intake, water retention or oedema in the body due to impaired renal function, increased blood pressure, and further damage to the kidney. However, in the real world, the relationship between decreased taste sensitivity and increased sodium intake is not straightforward. For example, sensitivity to salt taste is determined using very low concentrations, which in no way reflects the level of salt taste exposure in the food supply. Furthermore, at levels that reflect real-world salt taste exposure, individuals with CKD did not consider salt taste to be less intense than healthy adults in almost all studies in this review, suggesting that higher salt concentrations may not be needed. However, in our previous review on salt taste function and dietary intake [11], both salt taste sensitivity and the perceived saltiness of foods were poor predictors of sodium intake for a number of reasons, e.g., (i) high-sodium foods, e.g., cereals and breads, are consumed as staples but do not taste salty [43,44], and (ii) sodium at high concentrations is aversive and makes food unpalatable [45]. These factors may explain why salt taste thresholds and intensity did not consistently correlate with sodium intake in this review.

There were also some considerable limitations related to the dietary assessment of individuals with CKD in this review. First, a number of studies used spot- and 24 h urinary sodium excretion as a proxy estimation of sodium intake. This method has been shown to have high error rates [46,47], and its validity for use in individuals with CKD is questionable as this population clearly has impaired renal output. Thus, spot- and 24 h urinary sodium excretion measures may not provide accurate estimations of sodium intake. Second, low sodium intake advice is a standard intervention for individuals with CKD. Hence, dietary counselling may have influenced the dietary intake of individuals in these studies, instead of it being the consequence of taste changes. Third, it is widely known that individuals with CKD follow very restrictive diets, and they may eat very poorly on days when they are undergoing dialysis [48]. Therefore, the dietary intake reported in the studies included in this review may not reflect normal intake. Therefore, findings from studies that assessed dietary intake on days when participants attended their dialysis treatment should be considered carefully.

Our previous review indicated that salt taste hedonics are the most reliable predictors of salt taste intake in healthy adults, but the veracity of this in individuals with CKD was unknown [11]. In this review, while some studies reported significant differences between adults with and without CKD in terms of their liking and preference for salt taste, the evidence is not consistent, which could partly be due to the lack of standardised methods to examine salt taste hedonics (see Table 3). Moreover, two studies included a method that relied on participants’ familiarity with the foods in a questionnaire [39,42], and two other studies used salt water, which humans do not typically consume [31,38]. Only two studies used soups as test foods [29,40], which are actually consumed foods, yet these studies produced conflicting findings. It was noted that a study that reported no difference in salt taste preference had a very small sample size (*n* = 15 CKD), which results in limited statistical power to detect the difference between CKD patients and healthy controls [40].

While salt taste is often perceived as pleasant, the overall enjoyment of eating is often influenced by other tastes. In this review, there is evidence that taste sensitivity impairment in CKD was not limited to salt taste. In fact, taste impairment but also extended to sweet, sour, bitter, and umami tastes. Altered bitter taste function in CKD is likely to influence sodium intake, as salt is often used to mask the bitter taste of food [21]. Only one study included in this review assessed hedonic ratings towards these taste qualities, which is the aspect of taste perception that is the most likely to influence dietary behaviours [42]. That study reported that only the hedonic response to sweet foods was significantly lower in those undergoing HD but not CAPD [42]. Therefore, future studies should focus on understanding the liking and preferences of individuals with CKD for all tastes, and how well hedonic ratings translate into measurable dietary changes to inform dietary interventions in this population.

In the introduction section, we proposed that the alterations of several compounds in the blood and saliva of individuals with CKD may trigger taste disturbances. Several studies in this review measured these compounds. Serum and salivary zinc concentrations were associated with taste sensitivity [30,35] and intensity [31]; however, one study failed to find a relationship between zinc status and sensitivity [36]. Bitter taste intensity ratings were negatively correlated with salivary [31] but not serum urea [34]. Serum potassium concentration, which is often elevated in CKD, was also observed to be positively associated with the sensory ratings of potassium chloride (a salt tastant) [38]. If the relationships between blood or salivary compounds and taste function are confirmed, then an objective analysis of blood or saliva can be used in the future to identify individuals with CKD who are at risk of taste impairments.

Taste impairment in individuals with CKD was also reported to be more severe among those with diabetic nephropathy [30]. This was not too surprising as poorly controlled diabetes not only affects kidney function but also induces neuropathy. Our previous review demonstrated that individuals with diabetes, especially those for whom the condition is poorly controlled, also experienced taste impairment, potentially due to damage to the chorda tympani, greater petrosal, glossopharyngeal, and vagal nerves, which play important roles in human taste function [49]. The duration of CKD, but not the stage or the severity of CKD, was shown to influence taste function [28,41]. In other words, taste impairments are more common in individuals who have had CKD for an extended period of time.

Finally, CKD treatments were also implicated in taste dysfunction. First, dialysis was consistently shown to improve taste function (or reverse taste impairment) [23,28]; however, this was not influenced by how long the individuals with CKD had undergone dialysis [34]. This implies that individuals with CKD who undergo dialysis are less likely to experience taste impairments. The use of diuretics was also shown to be correlated with decreased salt taste sensitivity [30]. Although it has been hypothesised that diuretics block sodium channels (used to detect salt taste) [50] and increase zinc excretion [51,52,53,54] which, in turn, impair taste function, these hypotheses are yet to be confirmed.

This systematic review has several strengths and limitations. A strength of this review is the selection of studies that used objective salt taste function and perception measures. The synthesis of findings from the available studies provides more definitive evidence on the taste function of individuals with CKD. Knowledge generated from this review indicates that impaired taste sensitivity in CKD is less likely to have important dietary implications. Salt taste hedonics were largely unaffected. This review also has a number of limitations: i) a variety of methods were used to assess taste function and perception, which may partly explain the inconsistent findings, and makes the comparison of findings between studies difficult; ii) CKD is a broadly defined condition which encompasses various stages of disease and treatments, which likely influence taste function to different degrees, iii) all studies were observational in nature; hence, causation cannot be established, iv) limited studies examined salt taste hedonics, so a firm conclusion on this aspect cannot be drawn, v) few studies investigated the dietary intake of individuals with CKD, and the prescription of a low-sodium diet may interfere with the investigation between salt taste function and “actual” sodium intake; hence, the implications of a taste change for intake are likely to remain unknown, and vi) due to the prevalence of CKD, many studies were not able to include large sample sizes, which makes the ability to detect significant differences less likely. Given the limitations of this review, the findings need to be carefully considered before generalising and applying them to the broader population of CKD patients.

## 5. Conclusions

In summary, reduced sensitivity to salt taste was consistently observed in individuals with CKD, but salt taste intensity was not affected. Salt taste hedonics was not well investigated, and findings on this remain inconclusive. As taste sensitivity on its own is a poor predictor of dietary intake, individuals with CKD should be able to comply with a low-sodium diet, and the current available evidence does not support the call for a blanket strategy to manage salt taste changes in CKD.

## Figures and Tables

**Table 1:** Quality ratings of studies (*n* = 16) included in this systematic review using the Academy of Nutrition and Dietetics Evidence Analysis Checklist: Primary Research.

Author	Relevance ^1^				Validity ^1^										Quality Rating
	Q1	Q2	Q3	Q4	Q1	Q2	Q3	Q4	Q5	Q6	Q7	Q8	Q9	Q10	
Burge 1979 [27]	Y	Y	Y	N/A	Y	N	Y	N	U	Y	Y	U	Y	U	Neutral
Ciechanover 1980 [28]	Y	Y	Y	N/A	Y	U	Y	N/A	N	U	N	Y	Y	U	Neutral
Dobell 1993 [42]	N/A	Y	Y	N/A	Y	Y	Y	N/A	N	Y	N	Y	Y	N	Neutral
Fernstrom 1996 [37]	N/A	Y	Y	N/A	Y	Y	Y	N/A	Y	Y	Y	Y	Y	N	Positive
Fitzgerald 2019 [38]	Y	Y	Y	NA	Y	Y	Y	U	U	Y	Y	Y	Y	Y	Positive
Hurley 1987 [39]	Y	Y	Y	N/A	Y	Y	Y	N	N	Y	Y	Y	Y	Y	Positive
Kim 2018 [29]	Y	Y	Y	N/A	Y	Y	Y	N/A	N	Y	Y	Y	Y	Y	Positive
Kusaba 2009 [30]	Y	Y	Y	N/A	Y	Y	Y	N	N	Y	Y	Y	Y	Y	Positive
Manley 2012 [31]	N/A	Y	Y	N/A	Y	Y	Y	N/A	N	Y	Y	Y	Y	Y	Positive
Márquez-Herrera 2020 [32]	N/A	Y	Y	N/A	Y	Y	Y	N/A	Y	Y	Y	Y	Y	Y	Positive
McMahon 2014 [33]	N/A	Y	Y	N/A	Y	Y	Y	N/A	U	Y	Y	Y	Y	Y	Positive
Middleton 1999 [34]	Y	Y	Y	N/A	Y	Y	Y	N/A	U	Y	Y	Y	Y	N	Positive
Shephard 1987 [40]	Y	Y	Y	N/A	Y	Y	Y	Y	U	Y	Y	Y	Y	Y	Positive
Tavares 2021 [35]	N/A	Y	Y	N/A	Y	Y	Y	N	Y	Y	Y	Y	Y	Y	Positive
Vreman 1980 [36]	N/A	Y	Y	N/A	Y	Y	N	N/A	Y	Y	Y	Y	Y	N	Neutral
Yusuf 2021 [41]	N/A	Y	Y	N/A	Y	Y	Y	N/A	Y	Y	Y	Y	Y	N	Positive

^1^ Options for “Relevance” and “Validity” questions were Y (yes), N (no), U (unclear), or N/A (not applicable).

**Table 2:** An overview of the study design, participants’ characteristics, and measurements from the studies (*n* = 16) included in this systematic review.

Author, Year	Country	Study Design	CKD Participants ^1^	Controls	Salt Taste Measurements ^2^			Other Tastes ^2^	Dietary Intake ^2^
					DT/RT	Intensity	Hedonics		
Burge 1979 [27]	USA	Cross-sectional	*n* = 18 (9 Males 9 Females) Mean age = 46.5 years (range 17–65 years)	*n* = 10, age- and sex-matched with CKD Mean age = 45.8 years	RT	-	-	✓	-
Ciechanover 1980 [28]	Israel	Cross-sectional	Various stages of CKD (creatinine clearance 8–60 mL/min), *n* = 20, age range 25–77 years CKD with HD for >1 year, *n* = 23, age range 24–73 years	Controls consisted of: - Adults with various chronic diseases, *n* = 20, age range 35–80 years - Healthy controls, *n* = 22, age range 21–79 years	RT	-	-	✓	-
Dobell 1993 [42]	Australia	Cross-sectional	HD: *n* = 33 (19 Males 14 Females). Age: 19–59 years (*n* = 23); ≥60 years (*n* = 10), BMI: <20 kgm^−2^ (*n* = 6), 20–25 kgm^−2^ (*n* = 14), >25 kgm^−2^ (*n* = 13), time on dialysis: <1 year (*n* = 4), 1–5 years (*n* = 18), >5 years (*n* = 11) CAPD: *n* = 17 (9 Males 8 Females). Age: 19–59 years (*n* = 3); ≥60 years (*n* = 14), BMI: <20 kgm^−2^ (*n* = 3), 20–25 kgm^−2^ (*n* = 8), >25 kgm^−2^ (*n* = 6), time on dialysis: <1 year (*n* = 10), 1–5 years (*n* = 6), >5 years (*n* = 1)	*n* = 30 (14 Males 16 Females), adults with normal renal function. Age: 19–59 years (*n* = 10); ≥60 years (*n* = 20), BMI: 20–25 kgm^−2^ (*n* = 17), >25 kgm^−2^ (*n* = 13)	-	-	✓	✓	-
Fernstrom 1996 [37]	Sweden	Cross-sectional	*n* = 57, consisted of: - Pre-uremics: *n* = 31, age = 58.8 ± 13.2 years, BMI = 24.4 ± 3.7 kgm^−2^ - CAPD: *n* = 14, age = 49.6 ± 14.9 years, BMI = 23.3 ± 2.8 kgm^−2^, time on dialysis = 9.9 ± 11.1 months - HD: *n* = 12, age = 58.8 ± 12.6 years, BMI = 24.8 ± 5.2 kgm^−2^, time on dialysis = 16.9 ± 10.9 months	Healthy non-diabetic, *n* = 57, age, sex, BMI matched with CKD	RT	-	-	✓	-
Fitzgerald 2019 [38]	USA	Cross-sectional	CKD on maintenance HD, *n* = 17 (10 Males 7 Females) Age = 61 (range 23–87 years)	Control, *n* = 29 (13 Males 16 Females) Age = 32 years (range 21–61 years)	-	✓	✓	-	-
Hurley 1987 [39]	USA	Cross-sectional	CAPD, *n* = 10 HD, *n* = 10 Transplant, *n* = 10 Mean age of all groups 31.9–34.1 years	*n* = 10	-	✓	✓	-	✓ (3 d food records)
Kim 2018 [29]	Korea	Cross-sectional	CKD Stage 1–5, all non-dialysis, *n* = 436 (221 Males 215 Females), age = 55.5 ± 14.5 years - CKD Stage 1, *n* = 79 (39 Males 40 Females) - CKD Stage 2, *n* = 66 (34 Males 32 Females) - CKD Stage 3, *n* = 154 (87 Males 67 Females) - CKD Stage 4, *n* = 75 (35 Males 40 Females) - CKD Stage 5, *n* = 62 (26 Males 36 Females)	*n* = 74 (35 Males 39 Females) Age = 57.5 ± 11.5 years, GFR = 95.2 ± 13.1 ml/min/1.73 m^2^	DT & RT	-	✓	-	✓ (spot urinary sodium excretion)
Kusaba 2009 [30]	Japan	RCT (baseline data used)	*n* = 29 (19 Males 10 Females) Age: 62.9 ± 15.9 years, diabetic nephropathy *n* = 10	*n* = 11 (3 Males 8 Females) Age: 37.7 ± 8.62 years	DT & RT	-	-	-	✓ (24 h urinary sodium excretion)
Manley 2012 [31]	Australia	Cross-sectional	*n* = 30 (24 Males 6 Females) Age = 69.7 ± 14.2 years, GFR = 16.53 ± 5.21 mL/min	*n* = 5 (1 Male 4 Females) Age = 44.6 ± 10.3 years, GFR = 85.20 ± 6.50 mL/min	RT	✓	✓	✓	-
Márquez-Herrera 2020 [32]	Mexico	Cross-sectional	*n* = 75 (43 HD 32 CAPD) Age = 30 (range 26–43 years), 45% males	*n* = 112 Age = 22 (range 21–30 years), 30% males	RT	✓	-	✓	-
McMahon 2014 [33]	Australia	Cross-sectional	CKD stage 3–5, GFR = 33.1 ± 12.7 mL/min/1.73 m^2^, *n* = 91 (71 Males 20 Females) Age = 65.9 ± 13.5 years, eight = 89.3 ± 22.1 kg, BMI = 30.6 ± 6.4 kgm^−2^	*n* = 30 (14 Males 16 Females) Age = 55.2 ± 7.4 years, weight = 87.3 ± 19.0 kg, BMI = 29.7 ± 6.2 kgm^−2^	RT	✓	-	✓	✓ (40-item FFQ)
Middleton 1999 [34]	Australia	Cross-sectional	CKD on CAPD, *n* = 18 (11 Males 7 Females) Age = 52 ± 19.9 years, BMI = 23.6 ± 4.3 kgm^−2^, time receiving CAPD = 22 ± 14.3 months	*n* = 18 (11 Males 7 Females) Age = 52 ± 18.7 years, BMI = 24.1 ± 3.4 kgm^−2^	DT	-	-	✓	-
Shephard 1987 [40]	UK	Prospective	CKD on HD, *n* = 15 (9 Males 6 Females) Age = 47 years (range 17–62 years), time on dialysis = 2.8 years (range 0.25–6.7 years), time on reduced sodium diets = 0.25–10 years	*n* = 14, were staff at research institute, matched for age with CKD	-	✓	✓	✓	-
Tavares 2021 [35]	Brazil	Cross-sectional	CKD (non-dialysis), *n* = 21 (11 Males 10 Females) Age = 51.1 ± 7.1 years, BMI = 27.92 ± 7.07 kgm^−2^	*n* = 22 (10 Males 12 Females) Age = 49.8 ± 8.3 years, BMI = 28.48 ± 5.37 kgm^−2^	RT	-	-	✓	-
Vreman 1980 [36]	USA	Cross-sectional	CKD, *n* = 7 (all males), age: 58 ± 3 years CKD on HD, *n* = 26 (20 Males 6 Females), age = 41 ± 9 years	*n* = 48 (23 Males 25 Females), age = 65 ± 3 years	DT	-	-	✓	-
Yusuf, 2021 [41]	Nigeria	Cross-sectional	GFR < 60 mL/min/ 1.73 m^2^ *n* = 100 (56 Males 44 Females) Age = 46.3 ± 13.9 years (range 19–86 y), weight = 64.6 ± 12.6 kg	Healthy controls *n*= 100 (52 Males, 48 Females), age, sex matched with CKD Age = 43.4 ± 14.9 years (range 20–85 years), weight = 70.7 ± 13.2 kg	RT	-	-	✓	-

BMI—body mass index; CAPD—continuous ambulatory peritoneal dialysis; CKD—chronic kidney disease; DT—detection thresholds, GFR—glomerular filtration rate; HD—haemodialysis; RT—recognition thresholds. ^1^ Some studies did not indicate whether CKD participants were undergoing treatments such as HD and CAPD. ^2^ “-” indicates not assessed, while “✓“ indicates measurement taken in the studies.

**Table 3:** Findings on the salt taste function and perception of individuals with CKD.

(A) Detection Thresholds (*n* = 4)			
Study	Assessment Methods	Salt Taste Findings	Other Relevant Findings
Kim 2018 [29]	Salt taste thresholds were tested using NaCl solutions at 0.01, 0.025, 0.05, 0.075, 0.10,0.125, 0.15, 0.20, 0.3, 0.4, and 0.5% (11 stages). Beginning with the lowest concentration, participants swished and expectorated 1 test solution + 2 water controls in random order. The lowest concentration that was correctly detected twice consecutively was determined as DT.	DT was significantly higher in CKD Stage 3 and Stage 5 participants than controls (*p* < 0.05).	DT did not correlate with spot urinary sodium concentrations (proxy indicator of sodium intake).
Kusaba 2009 [30]	Sodium-impregnated taste strips (0%, 0.6%, 0.8%, 1.0%, 1.2%, 1.4%, and 1.6%) were placed in the mouth (in increasing order), where participants were asked if a taste was detected, and if yes, which. Test was repeated until participants correctly identified the taste twice.	Significantly higher DT in CKD (0.74 ± 0.21%) than controls (0.64 ± 0.08%) (*p* < 0.05). A total of 39% CKD vs. 18% controls had DT > 0.8%.	DT did not correlate with 24 h urinary sodium excretion in CKD (proxy indicator of sodium intake). Serum zinc negatively associated with DT in CKD (r = −0.67, *p* < 0.05). Significant higher DT in diabetic nephropathy (0.88 ± 0.28%) than non-diabetic CKD (0.66 ± 0.12%) (*p* < 0.05). Significantly higher DT in CKD treated with diuretics.
Middleton 1999 [34]	Multiple forced-choice solution presentation was performed in ascending order. Three cups (1 test solution + 2 controls) were presented in a pre-randomised order. Participants swished and expectorated the solutions and chose the one that was perceived to be different. NaCl solutions were presented in 11 concentrations ranging from 0.1 mmol/L to 31 mmol/L.	Significantly higher DT for salt taste was found in CAPD than controls (*p* = 0.001).	Significantly higher DT for bitter taste in CAPD than controls (*p* = 0.01). No differences were found for sweet and sour tastes. No significant correlations between DT and the length of CAPD, serum urea, and creatinine.
Vreman 1980 [36]	In ascending fashion, one of 14 NaCl solutions (ranging from 0.244 mM to 2000 mM) was presented together with 2 water controls in random order, and participants indicated which solution differed from water (3-alternative forced-choice method). Each NaCl solution was presented twice. The previous concentration presented to the subjects was determined as the threshold when both selections were incorrect. All participants were tested at least on 2 days, separated by 1 or more weeks. Mean DT was calculated.	DT was not significantly different between controls and CKD (with or without HD).	DT for sweet taste was significantly lower in CKD males than male controls. DT for sour taste was significantly lower in male CKD, and all CKD on HD. DT for bitter taste was significantly lower in male CKD on HD only. No significant correlation between tastes and blood zinc/copper concentrations in CKD HD.
**(B) Recognition Thresholds (*n* = 10)**			
**Study**	**Assessment Methods**	**Salt Taste Findings**	**Other Findings**
Burge 1979 [27]	Taste tests performed up to 30 min pre- and post- dialysis. Participants swished and expectorated NaCl solutions (0.005 M, 0.010 M, 0.020 M, 0.030 M, 0.040 M, 0.050 M, 0.060 M, 0.070 M, 0.080 M, 0.090 M, and 0.100 M), and were asked to describe the taste as being sweet, sour, bitter, salty, or no taste. RT was determined as the lower concentration when a taste was correctly identified twice consecutively.	RT for salt taste did not differ significantly between controls and CKD (both pre- and post-dialysis). It was noted that 4 CKD participants failed to recognise salt taste even at the highest concentration of 0.100 M.	RT for sweet and sour solutions of subjects pre-dialysis were significantly higher than those of the control subjects.
Ciechanover 1980 [28]	Salt taste solutions (5 NaCl concentrations: 0.030 M, 0.051 M, 0.079 M, 0.120 M, and 0.342 M) were dropped over the anterior dorsal surface of tongue. Following solution presentation, the tongue was retracted for 30 s, and participants were asked to swallow the solution and identify the taste.	RT for salt taste were significantly higher in both dialysed (*p* = 0.005) and non-dialysed (*p* = 0.01) CKD participants, but only in those <55 years.	Impairment also found in sweet, sour, and bitter tastes in CKD (dialysed and non-dialysed) adults < 55 years. In those >55 years, sour taste impairment was found in CKD (dialysed and non-dialysed). No correlation was found between severity of CKD and RT. Dialysis (pre- vs. post-) improved sour and bitter taste recognition, but in CKD < 55 years only. No association between RT and plasma zinc level.
Fernstrom 1996 [37]	A forced-choice ascending concentration series method measured DT. Participants swished and expectorated NaCl solutions at 0.01, 0.032, 0.10, 0.32, and 1.0 M, and recognition of taste was made on 6-point scales based on their ability to recognise the salt solutions.	RT was significantly higher (less sensitive) in pre-uremics and HD, but not in CAPD, than controls.	No association between salt taste function and age. No significant impairment in sweet and sour tastes was observed. Pre-uremics and CAPD were also less sensitive to bitter taste.
Kim 2018 [29]	Salt taste thresholds were tested using NaCl solutions at 0.01, 0.025, 0.05, 0.075, 0.10,0.125, 0.15, 0.20, 0.3, 0.4 and 0.5% (11 stages). Beginning with the lowest concentration, participants swished and expectorated 1 test solution + 2 water controls in random order. The lowest concentration that was correctly recognised twice consecutively was determined as RT.	RT was significantly higher in CKD Stage 3 than controls only.	RT did not correlate with spot urinary sodium level in CKD (proxy of dietary sodium intake).
Kusaba 2009 [30]	Sodium-impregnated taste strips (0%, 0.6%, 0.8%, 1.0%, 1.2%, 1.4%, and 1.6%) were used to assess salt taste thresholds. Taste strips in an increasing order were placed in the mouth and participants were asked if a taste was detected, and if yes, which taste it was. Test was repeated until participants correctly identified the taste twice.	Significantly higher RT in CKD (0.86 ± 0.26%) than controls (0.68 ± 0.14%) (*p* < 0.05). A total of 71% CKD vs. 27% controls had RT > 0.8%.	RT positively correlated with 24 h urinary sodium excretion in CKD (indicator of sodium intake) (r = 0.57, *p* < 0.01). No correlation between RT and serum zinc or diabetes status in CKD. Significantly higher RT in CKD treated with diuretics.
Manley 2012 [31]	Participants swished and expectorated 10 mL of salt solution (concentrations not reported) and identified taste by selecting one of 5 tastes or none if taste not perceived.	Salt taste recognition did not differ between CKD and controls (100% correct identification).	Significantly lower proportion of correct identification of sour, umami, and bitter tastes were found in CKD group.
Márquez-Herrera 2020 [32]	A taste perception test (TPT) of 5 taste qualities was developed in healthy controls and applied in CKD participants. Participants tasted the solutions (NaCl 0.5%) and were asked to identify.	Only CKD on HD were marginally (*p* = 0.06) less able to identify salty tastes.	CKD on CAPD were less able to recognise sweet and umami tastes (*p* < 0.05).
McMahon 2014 [33]	Participants were asked to identify the taste of a salt solution (200 mol/L NaCl) and rated the intensity on a VAS from 0 to 10.	Significantly lower proportion of CKD identified salt taste solution correctly (*p* = 0.01). This difference diminished when data were adjusted for age and gender (OR 3.9, 95%CI 0.8–18.7).	Sour was misidentified more frequently in CKD than control (*p* < 0.01), even after adjusting for age and gender differences (OR 4.8, 95%CI 1.8–13.0). 71% (*n* = 70) of CKD exceeded sodium intake recommendations (based on 40-item FFQ). A significantly larger proportion of CKD participants who met sodium intake recommendations identified salt and umami tastes correctly than those who exceeded recommendations (*p* < 0.01).
Tavares 2021 [35]	Three drops of NaCl solutions (4 concentrations 0.25, 0.1, 0.04, 0.016 g/mL) were placed on the tongue. Participants reported the perceived taste. The lowest NaCl concentration was recorded, and participants were also scored (range 0–4) based on the number of correctly identified solutions.	Recognition thresholds were not significantly different between CKD and controls (*p* = 0.590).	Significant correlations between plasma zinc and salt taste (r = 0.30, *p* = 0.048) and bitter taste (r = 0.49, *p* = 0.001) scores were found for all participants. No significant difference in sweet and sour taste function, but impaired bitter taste function in CKD (*p* < 0.001).
Yusuf 2021 [41]	Strips impregnated with 0.016, 0.04, 0.1, or 0.25 g/mL NaCl applied 1.5 cm from the tip of the tongue in increasing order. Taste function was obtained as the number of correctly identified tastes, with the highest scores given to the lowest NaCl concentration.	Significantly lower salt taste scores (less sensitive) in CKD (2.82 ± 1.1) than controls (3.7 ± 0.7) (*p* = 0.001).	Significantly lower scores in sour, sweet, bitter and total (all 4 taste qualities) taste scores in CKD than controls (all *p* = 0.001). Taste dysfunction was more severe with longer duration of CKD (*p* = 0.028) but not the stages of CKD (*p* = 0.629).
**(C) Intensity Ratings (*n* = 6)**			
**Study**	**Assessment Methods**	**Salt Taste Findings**	**Other Findings**
Fitzgerald 2019 [38]	Three salt solutions were used: NaCl 0.2 M, KCl 0.01 M, and Na_3_PO_4_ 0.0063 M. On haemodialysis days, participants swished and expectorated taste solutions for 10 s. After tasting each solution, participants reported perceived taste intensity.	Unadjusted intensity ratings were not significantly different (*p* = 0.73) between CKD and controls. After adjustment for ratings of water control, significantly higher intensity ratings were found for NaCl (*p* = 0.0018) and Na_3_PO_4_ (*p* = 0.017) in CKD than controls.	-
Hurley 1987 [39]	NaCl solutions at 0, 75, 150, 300, and 600 mmol/L concentrations were swished and expectorated, and the intensity ratings were assessed using modified magnitude estimation using a continuous scale 1 to 6.	No significant differences in intensity ratings between controls and HD, CAPD, and transplant.	-
Manley 2012 [31]	Participants swished and expectorated 10 mL of salt solution (concentrations not reported), identified taste, and then rated the perceived intensity on a 100 mm VAS from “water like” to “very strong”.	Salt intensity ratings did not significantly differ between CKD and controls.	Intensity ratings for sour and bitter were significantly lower in CKD than controls (both *p* < 0.05). Salivary bicarbonate was negatively associated with the intensity ratings of umami (r = −0.317, *p* = 0.002) and sour (r = −0.288, *p* = 0.03) tastes in CKD. Salivary urea was negatively associated with the intensity rating of bitter taste in CKD (r = −0.381, *p* = 0.04). Salivary zinc was negatively correlated with sweet taste intensity in CKD (r = 0.317, *p* = 0.02).
Márquez-Herrera 2020 [32]	A taste perception test (TPT) of 5 taste qualities was developed in healthy controls and applied in CKD participants. Participants tasted the solutions (NaCl 0.5%) and were asked to rate the intensity from 0 to 10 using a VAS.	No significant differences were found on salt taste intensity ratings between CKD and controls.	All CKD perceived sour taste to be less intense than controls (*p* < 0.05).
McMahon, 2014 [33]	Participants were asked to rate the intensity of the salt solution (200 mol/L NaCl) taste on a VAS from 0 to 10.	Intensity rating for salt taste did not differ between CKD and controls (*p* = 0.20).	Umami taste was rated significantly less intense in CKD than controls. CKD participants who met sodium intake recommendations rated umami and bitter tastes to be more intense (*p* < 0.01 and *p* = 0.03, respectively).
Shephard 1987 [40]	Participants tasted pea soup with 6 NaCl concentrations: 103, 155, 233, 349, 524, and 786 mg Na/l00 g soup in random order and rated intensity on a seven-category intensity scale from “No taste” to “Extremely strong”.	No significant differences in intensity ratings between CKD and controls.	No significant difference for sweet taste intensity. In CKD, salt taste intensity ratings were significantly higher after HD.
**(D) Hedonic Ratings (*n* = 6)**			
**Study**	**Assessment Methods**	**Salt Taste Findings**	**Other Findings**
Dobell 1993 [42]	A 88-item questionnaire including foods allowed on renal diets was used. Participants rated each food as “never tried it”, or how much they liked or disliked each food on a 9-point hedonic scale. These foods were then grouped into categories such as sweet foods, sour foods, salty foods, bitter foods (basic tastes), and other food groupings such as fruit, vegetables, red meat, cereal products, eggs, etc.	No significant difference in the mean liking of salty foods between HD (6.0 ± 0.2), CAPD (6.4 ± 1.0) and controls (6.1 ± 0.2) was found.	Liking of sweet foods was significantly lower in HD (6.0 ± 0.3) than control (7.1 ± 0.1) (*p* < 0.05). No significant differences were found in the liking of bitter and sour foods.
Fitzgerald 2019 [38]	Three salt solutions were used: NaCl 0.2 M, KCl 0.01 M, and Na_3_PO_4_ 0.0063 M. On haemodialysis days, participants swished and expectorated taste solutions for 10 s. After tasting each solution, participants reported their liking/disliking of the solutions.	Unadjusted liking ratings were not significantly different (*p* = 0.37) between CKD and controls. After adjustment for water control, significantly lower disliking score was found for NaCl (*p* = 0.045), KCl (*p* = 0.014), and Na_3_PO_4_ (*p* = 0.042) in CKD compared to controls.	Liking ratings for KCl were positively correlated with serum potassium levels in CKD (r = 0.57, *p* = 0.027).
Hurley 1987 [39]	Participants selected from a list of two-food combinations (one higher in sodium than another)	CAPD preferred salty foods more than controls (*p* < 0.01).	-
Kim 2018 [29]	Bean sprout soup containing 0.15% and 1.0% NaCl were used. Participants were instructed to add 1.0% NaCl soup to the 0.15% NaCl soup until a preferred salt concentration was reached. The final salt concentration was determined using a digital handheld salt tester. Test was conducted twice and sodium concentrations averaged.	Preferred salt concentration in soup was significantly lower in CKD Stage 5 (0.31 ± 0.09%) than controls (0.35 ± 0.12%) (*p* < 0.05).	Preferred salt concentration was positively associated with spot urinary sodium level (proxy of dietary sodium intake) (beta = 0.17, *p* = 0.022).
Manley 2012 [31]	Participants swished and expectorated 10 mL of salt solution (concentrations not reported) and rated their liking of taste solutions using a 9-point hedonic scale ranging from 1 “dislike extremely” to 9 “like extremely”.	Liking of salt solution did not differ between CKD and controls.	No significant differences in the liking of other taste solutions between CKD and controls. Salivary bicarbonate was negatively associated with umami taste liking in CKD (r = −0.307, *p* = 0.02).
Shephard 1987 [40]	Participants tasted pea soup with 6 NaCl concentrations, e.g., 103, 155, 233, 349, 524, and 786 mg Na/l00 g soup in a random order and rated on a 100 mm relative-to-ideal scale, which consisted of a 100 mm line anchored at the centre with the label “Just right”, at the left end with “Not nearly salty enough”, and at the right end with “Much too salty”.	No significant differences in preference between CKD and controls.	No significant difference was found for sweet taste preference between CKD and controls. In CKD, preferred salt concentrations in soup were significantly lower after HD.

BMI—body mass index; CAPD—continuous ambulatory peritoneal dialysis; CKD—chronic kidney disease; DT—detection thresholds, GFR—glomerular filtration rate; HD—haemodialysis; KCl—potassium chloride; NaCl—sodium chloride; Na_3_PO_4_—sodium phosphate; OR—odd ratio; RT—recognition thresholds.

## Data Availability

Not applicable.

## References

[1] Bikbov B., Purcell C.A., Levey A.S., Smith M., Abdoli A., Abebe M., Adebayo O.M., Afarideh M., Agarwal S.K., Agudelo-Botero M. (2020). Global, regional, and national burden of chronic kidney disease, 1990–2017: A systematic analysis for the Global Burden of Disease Study 2017. Lancet.

[2] Levey A.S., Coresh J. (2012). Chronic kidney disease. Lancet.

[3] Webster A.C., Nagler E.V., Morton R.L., Masson P. (2017). Chronic kidney disease. Lancet.

[4] Cheung A.K., Chang T.I., Cushman W.C., Furth S.L., Ix J.H., Pecoits-Filho R., Perkovic V., Sarnak M.J., Tobe S.W., Tomson C.R. (2019). Blood pressure in chronic kidney disease: Conclusions from a Kidney Disease: Improving Global Outcomes (KDIGO) Controversies Conference. Kidney Int..

[5] Ravera M., Re M., Deferrari L., Vettoretti S., Deferrari G. (2006). Importance of Blood Pressure Control in Chronic Kidney Disease. J. Am. Soc. Nephrol..

[6] Lewis J.B. (2010). Blood pressure control in chronic kidney disease: Is less really more?. J. Am. Soc. Nephrol..

[7] Garofalo C., Borrelli S., Provenzano M., De Stefano T., Vita C., Chiodini P., Minutolo R., De Nicola L., Conte G. (2018). Dietary Salt Restriction in Chronic Kidney Disease: A Meta-Analysis of Randomized Clinical Trials. Nutrients.

[8] Borrelli S., Provenzano M., Gagliardi I., Michael A., Liberti M.E., De Nicola L., Conte G., Garofalo C., Andreucci M. (2020). Sodium Intake and Chronic Kidney Disease. Int. J. Mol. Sci..

[9] Krikken J.A., Laverman G.D., Navis G. (2009). Benefits of dietary sodium restriction in the management of chronic kidney disease. Curr. Opin. Nephrol. Hypertens..

[10] McMahon E.J., Campbell K.L., Mudge D.W., Bauer J.D. (2012). Achieving Salt Restriction in Chronic Kidney Disease. Int. J. Nephrol..

[11] Tan S.-Y., Sotirelis E., Bojeh R., Maan I., Medalle M., Chik X.S.F., Keast R., Tucker R.M. (2021). Is dietary intake associated with salt taste function and perception in adults? A systematic review. Food Qual. Prefer..

[12] Tanaka M., Nishiwaki H., Kado H., Doi Y., Ihoriya C., Omae K., Tamagaki K. (2019). Impact of salt taste dysfunction on interdialytic weight gain for hemodialysis patients; a cross-sectional study. BMC Nephrol..

[13] Astback J., Fernstrom A., Hylander B., Arvidson K., Johansson O. (1999). Taste buds and neuronal markers in patients with chronic renal failure. Perit. Dial. Int..

[14] Matsuo R. (2000). Role of Saliva in the Maintenance of Taste Sensitivity. Crit. Rev. Oral Biol. Med..

[15] Kaushik A., Reddy S., Umesh L., Devi B.K.Y., Santana N., Rakesh N. (2013). Oral and salivary changes among renal patients undergoing hemodialysis: A cross-sectional study. Indian J. Nephrol..

[16] Anuradha B.R., Katta S., Kode V.S., Praveena C., Sathe N., Sandeep N., Penumarty S. (2015). Oral and salivary changes in patients with chronic kidney disease: A clinical and biochemical study. J. Indian Soc. Periodontol..

[17] Kho H.-S., Lee S.-W., Chung S.-C., Kim Y.-K. (1999). Oral manifestations and salivary flow rate, pH, and buffer capacity in patients with end-stage renal disease undergoing hemodialysis. Oral Surg. Oral Med. Oral Pathol. Oral Radiol. Endodontol..

[18] Tomás I., Marinho J., Limeres J., Santos M., Araújo L., Diz P. (2008). Changes in salivary composition in patients with renal failure. Arch. Oral Biol..

[19] Martins C., Siqueira W.L., Oliveira E., Primo L.S.d.S.G., Nicolau J. (2006). Salivary analysis of patients with chronic renal failure undergoing hemodialysis. Spéc. Care Dent..

[20] Manley K.J. (2014). Saliva composition and upper gastrointestinal symptoms in chronic kidney disease. J. Ren. Care.

[21] Keast R.S., Breslin P.A., Beauchamp G.K. (2001). Suppression of bitterness using sodium salts. Chim. Int. J. Chem..

[22] Kim S.M., Kim M., Lee E.K., Kim S.B., Chang J.W., Kim H.W. (2016). The effect of zinc deficiency on salt taste acuity, preference, and dietary sodium intake in hemodialysis patients. Hemodial. Int..

[23] Shepherd R., Farleigh C.A., Pryor J.S. (1986). Changes in Salt Taste in Dialysis and Their Relationship to Blood Constituents. Percept. Mot. Ski..

[24] Atkin-Thor E., Goddard B.W., O’Nion J., Stephen R.L., Kolff W.J. (1978). Hypogeusia and zinc depletion in chronic dialysis patients. Am. J. Clin. Nutr..

[25] Brennan F., Stevenson J., Brown M. (2020). The Pathophysiology and Management of Taste Changes in Chronic Kidney Disease: A Review. J. Ren. Nutr..

[26] Academy of Nutrition and Dietetics Evidence Analysis Manual: Steps in the Academy Evidence Analysis Process. https://www.andeal.org/evidence-analysis-manual.

[27] Burge J.C., Park H.S., Whitlock C.P., Schemmel R.A. (1979). Taste acuity in patients undergoing long-term hemodialysis. Kidney Int..

[28] Ciechanover M., Peresecenschi G., Aviram A., Steiner J.E. (1980). Malrecognition of Taste in Uremia. Nephron Exp. Nephrol..

[29] Kim T.H., Kim Y.H., Bae N.Y., Kang S.S., Lee J.B., Kim S.B. (2017). Salty taste thresholds and preference in patients with chronic kidney disease according to disease stage: A cross-sectional study. Nutr. Diet..

[30] Kusaba T., Mori Y., Masami O., Hiroko N., Adachi T., Sugishita C., Sonomura K., Kimura T., Kishimoto N., Nakagawa H. (2009). Sodium restriction improves the gustatory threshold for salty taste in patients with chronic kidney disease. Kidney Int..

[31] Manley K.J., Haryono R.Y., Keast R.S. (2012). Taste changes and saliva composition in chronic kidney disease. Ren. Soc. Australas. J..

[32] Márquez-Herrera R.M., Núñez-Murillo G.K., Ruíz-Gurrola C.G., Gómez-García E.F., Orozco-González C.N., Cortes-Sanabria L., Cueto-Manzano A.M., Rojas-Campos E. (2020). Clinical Taste Perception Test for Patients With End-Stage Kidney Disease on Dialysis. J. Ren. Nutr..

[33] McMahon E.J., Campbell K.L., Bauer J.D. (2014). Taste perception in kidney disease and relationship to dietary sodium intake. Appetite.

[34] Middleton R.A., Allman-Farinelli M.A. (1999). Taste Sensitivity Is Altered in Patients with Chronic Renal Failure Receiving Continuous Ambulatory Peritoneal Dialysis. J. Nutr..

[35] Tavares A.P.D.S.R., Mafra D., Leal V.D.O., Gama M.D.S., Vieira R.M.M.D.F., Brum I.D.S.D.C., Borges N.A., Silva A.A. (2021). Zinc Plasma Status and Sensory Perception in Nondialysis Chronic Kidney Disease Patients. J. Ren. Nutr..

[36] Vreman H.J., Venter C., Leegwater J., Oliver C., Weiner M.W. (1980). Taste, Smell and Zinc Metabolism in Patients with Chronic Renal Failure. Nephron Exp. Nephrol..

[37] Fernström A., Hylander B., Rössner S. (1996). Taste acuity in patients with chronic renal failure. Clin. Nephrol..

[38] Fitzgerald C., Wiese G., Moorthi R.N., Moe S.M., Gallant K.H., Running C. (2019). Characterizing Dysgeusia in Hemodialysis Patients. Chem. Senses.

[39] Hurley R.S., Hebert L.A., Rypien A.B. (1987). A comparison of taste acuity for salt in renal patients vs. normal subjects. J. Am. Diet. Assoc..

[40] Shepherd R., Farleigh C., Atkinson C., Pryor J. (1987). Effects of haemodialysis on taste and thirst. Appetite.

[41] Yusuf T., Raji Y.R., Lasisi T.J., Daniel A., Bamidele O.T., Fasunla A.J., Lasisi O.A. (2021). Effect of Chronic Kidney Disease on Taste Function: A Case Control Study among Nigerian. Indian J. Otolaryngol. Head Neck Surg..

[42] Dobell E., Chan M., Williams P., Allman M. (1993). Food preferences and food habits of patients with chronic renal failure undergoing dialysis. J. Am. Diet. Assoc..

[43] Magriplis E., Farajian P., Pounis G.D., Risvas G., Panagiotakos D.B., Zampelas A. (2011). High sodium intake of children through ‘hidden’ food sources and its association with the Mediterranean diet: The GRECO study. J. Hypertens..

[44] Anderson C.A., Appel L.J., Okuda N., Brown I.J., Chan Q., Zhao L., Ueshima H., Kesteloot H., Miura K., Curb J.D. (2010). Dietary Sources of Sodium in China, Japan, the United Kingdom, and the United States, Women and Men Aged 40 to 59 Years: The INTERMAP Study. J. Am. Diet. Assoc..

[45] Oka Y., Butnaru M., von Buchholtz L., Ryba N.J.P., Zuker C.S. (2013). High salt recruits aversive taste pathways. Nature.

[46] Dougher C.E., Rifkin D., Anderson C.A., Smits G., Persky M.S., A. Block G., Ix J.H. (2016). Spot urine sodium measurements do not accurately estimate dietary sodium intake in chronic kidney disease. Am. J. Clin. Nutr..

[47] Charlton K., Schutte A., Wepener L., Corso B., Kowal P., Ware L. (2020). Correcting for Intra-Individual Variability in Sodium Excretion in Spot Urine Samples Does Not Improve the Ability to Predict 24 h Urinary Sodium Excretion. Nutrients.

[48] Burrowes J.D., Larive B., Cockram D.B., Dwyer J., Kusek J.W., McLeroy S., Poole D., Rocco M.V. (2003). Effects of dietary intake, appetite, and eating habits on dialysis and non-dialysis treatment days in hemodialysis patients: Cross-sectional results From the HEMO study. J. Ren. Nutr..

[49] Tan S.-Y., Hack C., Yu C., Rennick I., Ohanian J., Dezan M., Mott N., Manibo R., Tucker R.M. (2021). Alterations in sweet taste function in adults with diabetes mellitus: A systematic review and potential implications. Crit. Rev. Food Sci. Nutr..

[50] Schiffman S.S., Lockhead E., Maes F.W. (1983). Amiloride reduces the taste intensity of Na+ and Li+ salts and sweeteners. Proc. Natl. Acad. Sci. USA.

[51] Golik A., Modai D., Weissgarten J., Cohen N., Averbukh Z., Sigler E., Zaidenstein R., Shaked U. (1987). Hydrochlorothiazide-amiloride causes excessive urinary zinc excretion. Clin. Pharmacol. Ther..

[52] Wester P.O. (2009). Urinary Zinc Excretion during Treatment with Different Diuretics. Acta Med. Scand..

[53] Mountokalakis T., Dourakis S., Karatzas N., Maravelias C., Koutselinis A. (1984). Zinc deficiency in mild hypertensive patients treated with diuretics. J. Hypertens. Suppl. Off. J. Int. Soc. Hypertens..

[54] Chiba M., Katayama K., Takeda R., Morita R., Iwahashi K., Onishi Y., Kita H., Nishio A., Kanno T., Saito T. (2012). Diuretics aggravate zinc deficiency in patients with liver cirrhosis by increasing zinc excretion in urine. Hepatol. Res..

